# Fatty Acid Profile in Goat Milk from High- and Low-Input Conventional and Organic Systems

**DOI:** 10.3390/ani9070452

**Published:** 2019-07-17

**Authors:** Annalaura Lopez, Mauro Vasconi, Vittorio Maria Moretti, Federica Bellagamba

**Affiliations:** Department of Health, Animal Science and Food Safety–Università degli Studi di Milano, Via Trentacoste 2, 20134 Milano, Italy

**Keywords:** goat milk, OBCFA, fatty acids, livestock production system, low-input, high-input

## Abstract

**Simple Summary:**

The nutritional composition of goat milk is the focus of an ongoing discussion regarding its possible consideration as functional food. Different livestock production systems can lead to a different nutritional composition of milk. Some classes of fatty acids, detected in favourable amounts, are considered important bioactive components of food because of their potential beneficial effects on human health. It is an interesting topic to consider in view of the current debate regarding the incidence of dairy products in the risk of human coronary heart diseases. In our study, we confirmed that a low-input (LI) production system in goats rearing, leads to a milk richer in favourable nutritional components compared to a high-input (HI) system. Moreover, comparing lipid profile of milk obtained under different rearing systems, a multivariate statistic approach allows for the discrimination between LI-organically certified, LI-non organically certified and HI-conventional goat milk samples. These results may contribute to enhance the characterisation of goat dairy products and could help raise the appreciation of consumers towards goat dairy products, thereby adding value to their market.

**Abstract:**

According to the knowledge that the composition in fatty acids of milk is related to the production system, we determined the fatty acid composition of goat milk yielded in three different Italian farms. Two low-input system farms; one organic (LI-O) and one conventional (LI-C), and one high-input system conventional farm (HI-C) were involved in the study. Significant differences were detected among the different groups considering the fatty acid pattern of milk. Fatty acids (FA) strictly related to the rearing system, such as odd and branched chain fatty acids (OBCFA), linoleic acid (LA, 18:2 n6), alpha-linolenic acid (ALA, 18:3 n3), elaidic acid (EA, 18:1 n9), total n6 and total n3 FA, were identified as the most significant factors in the characterization of samples coming from low- or high-input systems. OBCFA amounts were found to be higher (*p* < 0.05) in the LI-O milk (4.7%), followed by the LI-C milk (4.5%) and then by the HI-C milk (3.4%). The same trend was observed for Σn3 FAs, mainly represented by ALA (0.72%–0.81% in LI-O systems and 0.41% in HI-system), and the opposite for Σn6 FAs, principally represented by LA (2.0%–2.6% in LI-systems and 3.1% in HI-system). A significant (*p* < 0.01) discrimination among samples clusters coming from the different systems was allowed by the principal component analysis (PCA).

## 1. Introduction

The fatty acid (FA) composition and related factors variability in milk fat are recently renewed due to the impact of different FA classes in human nutrition, particularly, cis and trans monounsaturated fatty acids (MUFA), odd and branched chain fatty acids (OBCFA), conjugated linoleic acid (CLA) and polyunsaturated fatty acids (PUFA) [1,2]. Several studies aimed at discussing the linkage between animal diet and rumen microbiota, and the related effect on milk quality. The rumen bacteria population and mammary gland activity are the main responsible for biochemical mechanisms that include rumen biohydrogenation, mammary lipogenic and Δ9-desaturation in CLA and odd and branched chain formation [3,4,5]. Particularly the type of forage, forage to concentrate ratio (F/C), lipids supplementation and starch level, together with their interactions, significantly affect milk FA composition, including OBCFA. Bacterial OBCFA are major lipids of bacterial membranes [6,7]. A significantly different content of odd-chain (C15:0, C17:0, C17:1) fatty acids was reported in cow milk and cheese samples from a different pasture vegetation type, contributing in the characterization and protection of typical dairy products [8,9]. Branched chain fatty acids (BCFA), iso and anteiso forms relating to the methyl-group located on the penultimate carbon and on the antepenultimate carbon of the carbon chain, represent a lesser component of milk (about 2%–3% of total fatty acids). However, they are recognised as important bioactive components since their positive role in gastrointestinal microbial ecology and potential anti-cancer activity [10,11,12,13]. Cytotoxicity of these fatty acids might be compared to that of CLA to which much more attention has been spent, despite milk BCFA being more represented than milk CLA. Recently, the contribution of BCFA from various food, prominently featured in the American diet, has been estimated. These studies have shown that BCFA may have a beneficial effect on proper gut functions; thus, their intake becomes relevant for human health [11]. Grazing represents a right approach to improve healthful nutritional quality of milk lipid composition in ruminant species. Goat milk products seem to be enriched in OBCFA when compared to cow milk products [14], and this could be one of the criterions useful in enhancing the appreciation of consumers toward goat products, and to take them into account as possible functional foods. In goat milk, the OBCFA content was significantly affected by lipid supplementation and its interaction with forage levels [15,16]. Moreover, different percentages of diet concentrate affect the relative amount of C13:0, *iso*-C15:0, and *iso*-C16:0 in goat fat milk [17]. Comparisons of organic and conventional farming system characterized by grazing and a reduced amount of conserved forage generally show a higher proportion of nutritionally favourable FA such as PUFA n3, rumenic acid and branched FA [18,19,20,21,22]. Diets rich in starch or a decrease in F/C ratio and neutral detergent fibre (NDF) content promote the growth of amylolytic and go to detriment of cellulolytic bacteria with a consequent reduction of iso fatty acids in milk fat [5]. Generally, the conventional livestock production system adopted in dairy goat breeding in Italy, particularly in small mountain farms, is considered as semi-intensive. This could be the reason for the higher amount of OBCFA detected in goat products. It is interesting to determine if any difference or similarities exists among milk yielded in farms managed under different conditions. Particularly, in many goat farms, which we can consider as “low-input-system farms”, the production system is not so different to that used in organic-certified farms, and this could lead to a similar nutritive and functional quality of yielded milk, even if it is not certified as organic [23]. Thus, the aim of this work was to evaluate the impact of different production systems in Italy on milk fatty acids composition of goat milk, with a particular focus on OBCFA. 

## 2. Materials and Methods

### 2.1. Animals, Housing and Feeding

The experiment involved three goat farms from the end of March until October 2017. Each farm was considered for different peculiarities distinguishing its livestock production system. With high-input (HI), authors refer to the conventional, typically intensive, dairy goat rearing system in Italy, in which animals are always reared in the barn and the diet is mainly based on concentrates, conserved forage and a low F/C ratio all the year around. A conventional lowland farm with Saanen breed goats (HI-C) was selected to represent the HI production and feeding system (HI-C). In the HI-C farm, goats were fed by local ryegrass hay offered *ad libitum*, alfalfa hay offered once a day (about 500 g/d) during the first period of lactation and commercial feed, consisting of a mixture of flaked and flour cereals, distributed individually twice a day (totally 1200 g/d) during milking. Two farms were considered representing the low-input (LI) production system. In the LI-system, goats are mainly allowed to graze. In the barn, their diet is integrated with conserved forage and concentrates, with a higher F/C ratio. A conventional mountain farm (altitude, 980 m) with Alpine breed goats, represented the not-organically-certified LI breeding system in our study (LI-C). In the LI-C farm, goats grazed depending on favourable climatic conditions and other feed supplements were also included in the diet. In more detail, goats were fed *ad libitum* with local polyphite grass hay (first harvest), alfalfa hay distributed once a day after the morning milking (about 500 g per goat per day) and concentrates (commercial mixtures) distributed twice a day during milking (totally 1000 g per goat per day). Finally, an organic Alpine breed goat farm (LI-O) was selected representing the organic-certified [24] system, in which, in addition to feeding, further organic-farming standard must be observed (LI-O). In this farm, lactating goats were systematically and daily grazed from spring to autumn (March–October); the access to fresh grass was controlled, starting after the morning milking and ending around noon (about 4 hours/day). Forage consisting of alfalfa hay (1000 g per goat per day) and polyphite grass hay (500 g) was distributed twice a day, firstly in the morning before grazing fresh pasture and after the second milking in the afternoon grains. A mixture of organically certified maize and barley grains (3:1, respectively), formulated in-farm, was supplied twice a day during milking (totally 800 g/d). In all three farms goats had free access to water and to salt integrators; no additional lipids were supplemented. Feeding (including access to pasture) and milk yield and composition data were recorded by information collected by farmers. The average daily feed intake per goat was evaluated on the basis of dry matter intake (DMI) prediction models for lactating goats reported by Pulina et al. [25], taking into account the goat’s average size and milk yield for the lactation period involved in the study. Estimated intakes were calculated by fodder analysis of feedstuffs dry matter [26].

### 2.2. Sampling 

All farms had similar proportions of goats in lactation at all sampling dates (42, 39 and 45 in HI-C, LI-C and LI-O farms, respectively). Goats were machine milked twice a day in all farms. Bulk tank raw milk samples were sampled twice a month, from March (4 ± 1 week of lactation) to October, from two consecutive milking, corresponding to cheese making in farm. A total amount of 37 milk samples were stored at minus 20 °C until the analysis of fatty acid composition. The measurement of fat, protein and lactose were assumed from monthly official controls, determined by near infrared spectrophotometry [27]. 

### 2.3. Analytical Procedures

To determine the fatty acids composition of milk, fat was extracted according to the method of Folch et al. [28]. Lipids were extracted from 10 mL of milk by chloroform methanol 2:1. Extracted milk fat was quantified and an aliquot (about 40–50 mg) was used for fatty acid composition analysis. Fatty acids were determined as methyl ester, prepared by base catalysed methanolysis of glycerides as described by Christie [29]. Lipids were dissolved in 1 ml of diethyl ether, then 50 µL of methyl acetate and 100 µL of 1 M sodium methoxide in methanol were added. The reaction was stopped after 5 min at room temperature by adding 50 µL of an oxalic acid in diethyl ether saturate solution. After centrifugation at 1500 g for 5 min, 200 µL of upper layer of solution was used directly for gas chromatography (GC) analysis. The injector was set in split mode (1:100 split ratio) at a constant temperature of 250 °C and 1 µL sample was injected. Fatty acid analysis was carried out on an Agilent gas-chromatograph (Model 6890 Series GC, Agilent Technologies, Santa Clara, CA, USA) fitted with an automatic sampler (Model 7683, Agilent Technologies, Santa Clara, CA, USA) and FID detector (Agilent Technologies, Santa Clara, CA, USA). The carrier gas was helium with a flow rate of 1.0 mL min-1 and an inlet pressure of 16.9 psi. A TRACE™ TR-FAME column (60 m length, 0.25 mm i.d., 0.25 µm film thickness; Thermo Fisher Scientific, Waltham, MA, USA) was used to separate fatty acid methyl esters. The oven temperature program for separation started with an isotherm of 6 min at 50 °C, then the temperature increased at a rate of 10 °C min^−1^ until 170 °C and kept at this temperature for 30 min. Afterwards the temperature was increased from 4 °C min^−1^ to the final temperature of 220 °C and hold for 20 min. 

Individual fatty acids methyl esters were identified by comparing sample peak retention times with standard mixtures (Supelco 37 FAME Mix, Supelco, Bellafonte PA, USA) and pure standard methyl esters from Sigma- Aldrich (Sigma-Aldrich, Saint Louis, MO, USA, cat n.CRM 47791) and expressed as percentage of total fatty acids. The identification of branched chain fatty acids was determined by preparing methyl esters from standards available (Sigma-Aldrich, Saint Louis, MO, USA), then analysed under the same instrumental condition. A gas-chromatographic correction factor has been applied to take into account the lower response of the flame ionization detector to the molecules with a lower number of carbon atoms for 4:0, 6:0, 8:0, 10:0 and 12:0 [30].

### 2.4. Statistical Analyses

The evaluation of different farming systems was calculated by the analysis of variance. Normal distribution (Shapiro-Wilk test) and homogeneity of variance (Levene test) were confirmed and comparison between means was performed by the ANOVA test when the normality and homoscedasticity assumptions were confirmed, and the Welch ANOVA F test when the assumptions were not confirmed. The Student Newman-Keuls was used as the post Hoc test for comparison of the means among different farming systems. Significance was declared at *p* ≤ 0.05. Multivariate analysis (principal component analysis, PCA) was applied, taking into account all fatty acids of milk sampled as graphical projection technique, in order to study the distribution of samples in a two-dimensional space and to establish if any separation between different groups (LI-O, LI-C, HI-C) was feasible using measured variables. The statistical procedure was performed using JMP Pro 14 (SAS Institute Inc., Cary, NC, USA). Data in the tables are reported as mean values ± standard deviation.

### 2.5. Ethical Approval

This article does not contain any experimental practice performed on animals by the authors. Only raw bulk milk sampling, considered a routine practice in farm, was performed in order to develop the present research. No biological material was collected. Authors guarantee that in the three farms involved in the study all the applicable guidelines for animal welfare established by harmonised EU rules were followed. No approval by the institutional ethics committee was requested by University of Milan for this kind of research.

## 3. Results 

Table 1 discloses goat feed intake and milk proximate composition of farms involved in the study. Proximate composition did not show significant differences between the three groups, with the exception of protein concentration, which slightly increased in HI-C farm (*p* < 0.05).

The results obtained from fatty acid analysis are reported in Table 2, expressed as g/100g of total FA. 

Detected fatty acids were divided into four groups according to their chemical structure. For each group, the sum (Σ) was made. Particularly, for the OBCFA group we calculated the amount of branched-chain fatty acids (Σ BCFA), linear odd-chain fatty acids (Σ OCFA) and the total amount of both groups (Σ OBCFA). Oleic acid was found in higher concentrations in LI-systems samples (21.1% in the conventional farm and 18.9% in the organic farm) than in the HI-system samples (17.4%). Elaidic acid (EA) and linoleic acid (LA) were found to be higher (*p* < 0.05) in the HI farm milk (0.48% and 3.1%, respectively), followed by the LI-C farm milk (0.41% and 2.6%, respectively) and, then, by the LI-O milk (0.29% and 2.0%, respectively). Alpha-linolenic acid (ALA) showed differences (*p* < 0.05) between the LI farms milk (0.72% in the organic farm and 0.81% in the conventional farm) and the HI farm milk (0.41%). PUFA of the n3 series were found in higher (*p* < 0.05) amounts in LI-O milk (1%) and LI-C milk (1.1%) than in HI-C milk (0.6%). On the contrary, n6 PUFA were significantly higher (*p* < 0.05) in HI-C milk (3.8%), followed by LI-C milk (3.4%) and, then, by LI-O milk (2.6%). Consequently, the n6/n3 ratio showed differences (*p* < 0.05) between LI groups, with values of 2.6 in the organic farm and 3.4 in the conventional farm, and the HI group, in which it reached a value of 3.8. Long chain n3 PUFA, EPA and DPA, resulted in higher values (*p* < 0.05) in the LI-systems than in the HI-system. DHA (22:6 n3) was detected only in trace and in a small number of samples. Significant differences (*p* < 0.05) were found among the three groups in the whole content of OBCFA, which ranged from 4.7% in the LI-O farm to 3.9% in the HI-C farm, and with the LI-C farm it reached an intermediate value of 4.4%. Particularly, branched fatty acids iso14, iso16 and the linear-odd fatty acid 15:0 showed different (*p* < 0.05) amounts in LI-O, LI-C and HI-C samples. Branched-chain were the most representative, reaching values of 2.6%, 2.4% and 2.2% in LI-O, LI-C and HI-C, respectively, while linear-odd chain fatty acids ranged from 2.1% in LI-O milk to 1.7% HI-C milk.

**Figure 1:**
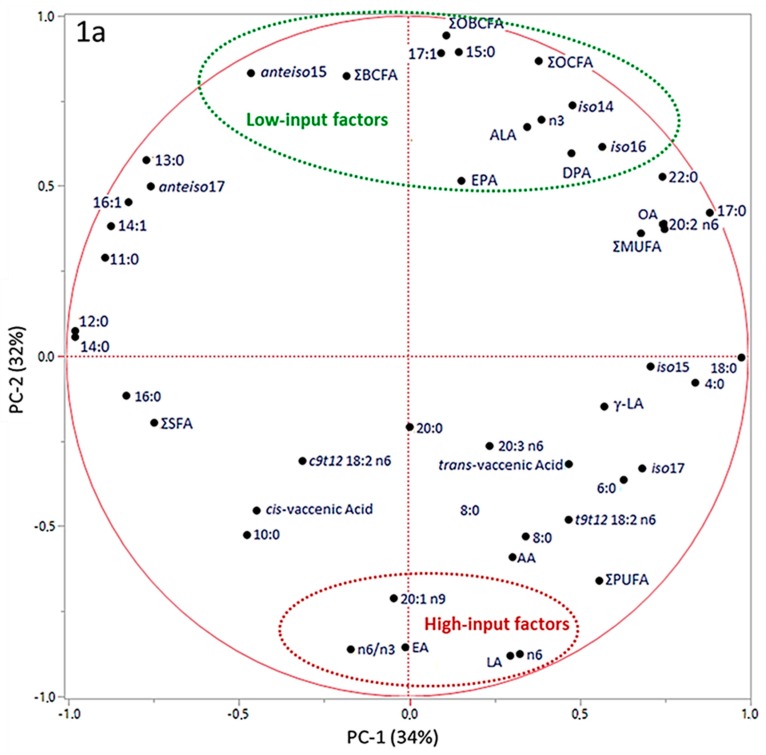

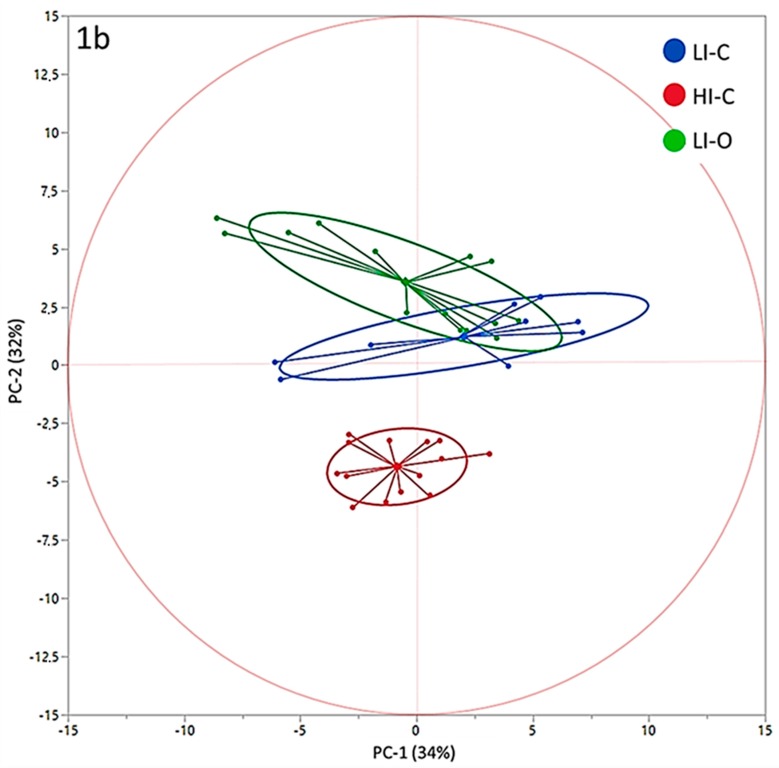
Principal Component Analysis Loadings Plot (**a**) and Scores Plot (**b**). a, b, c = values in the same row that have different superscript are significantly different at *p* ≤ 0.05, ANOVA and Student-Newman-Keuls post-hoc test.

In Figure 1, the results obtained by principal component analysis (PCA) of data are reported.

**Figure 2:**
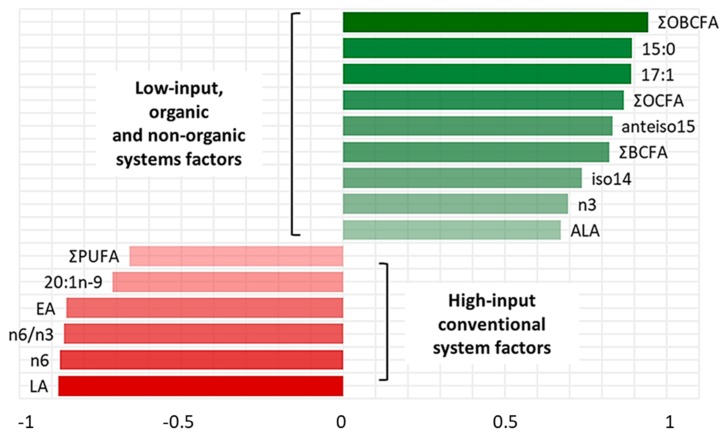
Principal Component-2 eigenvectors of factors considered as representative of the low-input and the high-input systems. Variables with absolute values of their eigenvectors equal or greater than 0.6 have been chosen. Factors with the higher absolute values of their eigenvectors are associated with a higher influence on data variability.

PC-1 and PC-2 have been chosen as coordinates on the x- and y- axes, accounting for 34% and 32% of total variance in data distribution, respectively. This combination of PCs has been chosen as it allowed for the best separation of the groups. In the correlation loadings plot (Figure 1a) we can see that OBCFA, EA, LA, ALA, n3 FA, n6 FA and n6/n3 ratio could be identified as factors that more significantly contributed to the variance among samples, by PC-2 vector’s direction. The scores plot (Figure 1b) shows three clusters, with the average point, variability within the groups and density ellipses set at 0.68 (mean ± 1 standard deviation) for each group. PC-2 allowed a discrimination among samples coming from the three different farms. Both LI-O and LI-C groups were distributed in the area of the two-dimensional space where the “low-input factors” (green) are positively correlated with PC-2, in line with the higher values found in these samples by chemical analyses. On the contrary, HI-C samples are more characterized by the “high-input factors”, which presented negative loadings on PC-2. In Figure 2, the most influencing factors on clusters discrimination and their eigenvectors on the second component of PCA are reported.

The factors taken into account could be divided in two groups, according to their positive or negative relation with PC-2. Variables selected in this model were total OBCFA, n3 and ALA, which showed the overall maximal values in LI-systems samples, while higher levels of LA, n6, EA, 20:1n9 and PUFA content were observed in the HI-system samples. In Table 3, results of the analysis of variance on PC-1 and PC-2 are reported. The ANOVA of the PC scores revealed that whereas PC-1 did not allow us to discriminate different samples, PC-2 effectively separated the three groups (*p* < 0.01) according to the livestock production system. 

## 4. Discussion

No significant differences were found in the proximate composition of milk samples coming from the different farms, except for the slight differences in protein amounts. In all three groups, fatty acids showed the characteristic fatty acid pattern of caprine milk and values of fatty acids from 6:0 to 10:0 agree with values previously reported in literature [17,31,32]. Fatty acids from 4 to 8 carbon atoms were not found to be significantly different in milk obtained from different farms and confirmed the previously observed results [17], proving that such differences in production systems did not modify volatile fatty acids proportion in goat milk. The proportion of oleic acid in milk is controlled by its plasma uptake and partly from desaturation of stearic acid by mammary Δ9-desaturase [33]. The desaturation ratio 18:1 c9/18:0 in the mammary gland is influenced by the diet, since the increase in availability of either PUFA or *trans*-FA inhibits the Δ9-desaturase. Consequently, the proportions of oleic acid in goat milk decrease with the increasing percentage of concentrate [17]. According to this, we detected the lowest values for oleic acid in samples of HI-C farm, in which goats were fed with the higher amount of concentrates (50% of DMI). Even the concentration of *trans*18:1 isomers (*t*9 + *t*11) is related to the percentage of concentrate in the diet, and it increases when the concentrate proportion is higher [34]. Rumen microbial population is considered directly involved in this process, since *trans*18:1 isomers are intermediary products of ruminal biohydrogenation of the dietary PUFA [35]. The main factor that affects the biohydrogenation rate is the percentage of the concentrate in the diet [36]. In literature, vaccenic acid is the major component (about 36.2%) of total *trans*18:1 isomers [37]. In our study, vaccenic acid (18:1 t11) represented 70% to 80% of the detected *trans*18:1 isomers, without differences among different farms. However, the lowest amount of VA was detected in LI-O milk and the highest in HI-C and LI-C milk. Elaidic acid (18:1 t9) relative content resulted different (p<0.05) in the three groups of milk samples. The relative percentages of EA detected matched with the results obtained by Serment et al. [17], who reported an effect of the percentage of concentrate on the fatty acid profile in milk goats that produced a relative amount of 0.3% of EA in a lower concentrate diet versus an amount of 0.4% of EA in a higher concentrate one. These results are consistent with the knowledge that a decrease in the fiber content (or an increase of concentrate) in the goat daily ration would lead to a higher content of the *trans*18:1 fatty acids in milk (except 18:1 t11) [36,38].

In ruminants, the unsaturated fatty acids are metabolized by microorganisms in the rumen and undergo biohydrogenation and double-bond migration to yield a mixture of structural isomers (cis-trans isomers and positional isomers) [39]. PUFA quantities in ruminant milk generally increase with PUFA dietary intake, even if the transfer efficiency of PUFA from the diet to milk is low because of the biohydrogenation process that occurs in the rumen [33]. In our research, similar amounts of PUFA were detected, with a difference (*p* < 0.05) between samples coming from the high-input system (4.41%) and samples coming from the low-input systems (4.43% in the conventional one and 3.6% in the organic one). It is known that the fresh forage intake increases dietary PUFA supply [23,40,41,42]. However, some studies [43,44] have reported that a high amount of concentrates in diet can cause a decrease in biohydrogenation processes, with a consequent increase of PUFA amounts. Moreover, in Table 3 it is shown that the higher amount of PUFA in HI-C milk is mainly due to the higher amount of n6FA in these samples. The concentration of linoleic acid, the most representative FA among PUFA, was higher in HI-system milk compared to LI-systems milk. These results agree with values reported in literature, specifically in the absence of lipids added to diets, as the proportion of LA on goat milk FAs is between 2% and 3% [15,17,32,36]. Generally, all or most of LA in milk fat comes from dietary LA that escapes from rumen biohydrogenation activity, and its transfer to milk is related to the amount of this fatty acid that is ingested [45]. Most likely, we found higher LA values in HI-system milk because of the higher intake of concentrates by goats reared in this farm, according to the fact that by decreasing the F/C ratio the LA concentration increases [36]. Alpha-linolenic acid and long chain n-3 series in goat milk are influenced by a fresh grass-based diet that is a good source of ALA. Consequently, pasture induces an increase in ALA in ruminant milk [46]. In our results, ALA content was found to be significantly higher in LI-systems milk than in HI-system milk. The higher amount of ALA in LI-systems milk could be explained by the fact that goats at these farms had access to pastures normally enriched in ALA, and that were fed with a natural grassland hay, which had in the past showed a positive effect on ALA content in goat milk [36]. Furthermore, forage is rich in ALA, whereas cereals contain higher amounts of LA [45]. HI-C goats were fed with the highest amount of concentrates and the lowest F/C ratio. Similar amounts below or around the 1% of ALA were reported by other authors in milk from Alpine goats fed a hay-based diet at a low percentage of concentrate and without lipid supplementation [17,31,47]. In the milk of Saanen goats fed with a high F/C ratio diet, Mele et al. [16] showed a presence of ALA (0.49%) comparable to our results. In addition to total PUFA and ALA, CLA (LA-conjugated isomers) are generally considered as markers of fiber intake in ruminants [36]. CLA c9t11, which is the most representative of total CLAs, shows higher concentrations when the proportion of fresh grass intake increases [48]. Unfortunately, with our analytical equipment, provided with a 60 m TR-FAME ™ column, we were able to separate two LA-non conjugated isomers,18:2 t9t12 and 18:2 c9t12, but we had the coelution of the CLA c9t11 with 20:0. For this reason, the amount of 20:0 reported in Table 2 can include also CLA c9t11. EPA and DPA relative content in Alpine goat milk resulted in higher values than the ones reported by Bernard et al. [47]. The author studied fatty acids trend in Alpine goat milk, reporting values of 0.07% for EPA and 0.12% for DPA in milk goats fed with natural grassland hay. Differently, the relative amount of EPA we detected in HI-C milk (0.06%) was lower than the amount reported by other authors [16] in Saanen milk (0.11%). Since PUFA are not synthesized by tissues in ruminants, their concentration in milk is positively related to the dietary amount and to the decrease of rumen hydrogenation activity, such as a high forage/concentrate ratio. According to this, we found the highest amount of EPA and DPA in milk samples coming from the two LI-systems farms.

In our study, OBCFA showed differences (*p* < 0.05) between the HI-farm milk and the two LI-farms milk. In previous studies [4] linear odd-chain fatty acids were reported as the majority of ruminant milk OBCFA, followed by anteiso-fatty acids. In the present work we found the higher values in branched-chain fatty acids. The most representative were *anteiso*15:0 and *anteiso*17:0, which reached the higher values in LI-O milk in both cases (0.7% and 0.8%, respectively), then followed by *iso*-forms. These results follow the trend observed by Alonso et al. [32] who found that the most important OBCFA in quantitative terms in goat milk were the *iso*15:0 and *anteiso*15:0, *iso*17:0 and *anteiso*17:0 and *iso*16:0. We found the main differences in *iso*14:0, 15:0 and *iso*16:0 fatty acids, with the higher amount in LI-O milk, followed by LI-C milk and by HI-C milk. These differences may be relevant by a nutritional point of view. Indeed, 15:0 levels in subcutaneous adipose tissue and serum have been used as markers of intake of ruminant fat by humans [49,50,51]. Meanwhile, it is known that *iso*14:0 and *iso*16:0 levels in milk fat are related to the rumen functionality, showing a positive association with the presence of cellulolytic bacteria and decreasing when diets rich in starch are supplied to animals [5,52,53]. On the contrary, increasing the forage/concentrate (F/C) ratio in the diet resulted in a higher proportion of milk OBCFA, even if the effects of variation in the dietary F/C ratio on OBCFA are not uniform over all studies found in literature [5]. In the present study, we found higher levels of total OBCFA in goats that had access to grazing and were fed with the higher amount of forages. These changes in milk OBCFA concentration might reflect the equilibrium in the rumen bacterial populations induced by the differences in dietary F/C ratio and by the quality of the fiber supplied (fresh grass vs conserved forage).

Also, the n6/n3 ratio, considered as a healthy balance index, was evaluated. We obtained a difference (*p* < 0.05) between samples collected in the LI farms (both organic and conventional) and samples collected in the HI farm. The lower amounts of n6/n3 were detected in the LI-O milk, followed by LI-C milk and then by HI-C milk. A lower n6/n3 ratio is indicative of a forage-based diet [21] and this is consistent with the fact that in LI farms goats were fed with a higher amount of forage. Values obtained in this research for the LI-systems products display lower values for n6/n3 ratio than values reported in literature for goat milk (5.0) [54], suggesting a more favorable composition of products originated by these rearing systems.

According to our discussion regarding fatty acids composition in the different groups, variables associated with positive values of PC-2 eigenvectors (Figure 2, in green) could be considered as markers of the low-input systems, both organic and non-organic, mainly related to the higher F/C ratio of the supplied diet and to the access to pastures. On the contrary, variables associated with negative values of PC-2 eigenvectors (Figure 2, in red) could be considered as markers of the high-input system, characterized by a lower F/C ratio in the diet and by the exclusive use of conserved forages supplied in the barn. Thus, even if a partial overlapping between LI-O and LI-C is observable in the scores plot (Figure 1b), the analysis of variance of PC eigenvalues (Figure 2) revealed that the combination of original variables obtained with PCA on the first two components allowed us to identify three different clusters. It suggests that the total pattern of milk fatty acids is significantly influenced by the considered production systems, more so than single fatty acids. Moreover, LI-O and LI-C goats milk composition showed the largest variability of data (Figure 1b), putatively linked to seasonal variations and to the quantity and the quality of feed in pasture-based systems, compared with the HI-C milk farm, where the diet had very few variation all over the research time.

## 5. Conclusions

The present study have mainly demonstrated that goat milk from farms managed under a low-input rearing system showed differences if compared to milk from a high-input system farm. A multivariate statistical technique supported results found by analytical means. Differences were particularly detectable between the LI and the HI farming systems, but few differences were found between the two LI-system farms. The differences were mainly imputable to different amounts of some fatty acids, primarily OBCFA, n3FA, n6FA, ALA, LA and EA, selected by the Authors as factors related to the farming system. 

## Figures and Tables

**Table 1:** Feed intake and milk yield (per goat per day) and composition in LI-O, LI-C, HI-C. Milk composition data are presented as mean ± standard deviation for each farm.

Item	Farm									
	LI-O Low Input, Organic				LI-C Low Input, Conventional			HI-C High Input, Conventional		
	C^1^	AH ^2^	PGH ^3^	P^4^	C ^1^	AH ^2^	PGH ^3^	C ^1^	AH ^2^	RH ^5^
supplied (g/day)	800	1000	500	Ad libitum	1000	500	Ad libitum	1200	500	Ad libitum
g/Kg DM	297.1	362.1	183.1	-	423	215.6	-	488.1	207.7	-
F/C ratio	70/30				60/40			50/50		
Milk yield(g/day)	2.55 ± 0.5				2.50 ± 0.6			2.60 ± 0.5		
Fat (%)	3.2 ± 0.7				3.3 ± 0.5			3.6 ± 0.55		
Protein (%)	3.2 ± 0.3 ^a^				3.3 ± 0.3 ^a^			3.7 ± 0.4 ^b^		
Lactose (%)	4.4 ± 0.3				4.4 ± 0.3			4.6 ± 0.15		

^1^ C - concentrates; ^2^ AH - alfalfa hay; ^3^ PGH - polyphite grass hay; ^4^ P - pasture; ^5^ RH - ryegrass hay. ^a,b^ = mean values for each group within a row with unlike superscript letters were significantly different (*p* < 0.05).

**Table 2:** Fatty acids (g/100g of total FA) of bulk goat milk samples. Data are expressed as mean ± standard deviation.

Fatty Acid	Low Input-Organic (n = 14 *)		Low Input -Conventional (n = 9 *)		High Input-Conventional (n = 14 *)	
Saturated Fatty Acids						
4:0, Butirric Acid	2.0	±0.30	1.9	±0.29	1.9	±0.17
6:0, Caproic Acid	2.0	±0.28	1.9	±0.17	2.1	±0.14
8:0, Caprylic Acid	2.7	±0.36	2.6	±0.19	2.9	±0.16
10:0, Capric Acid	10.0 ^a^	±0.77	9.3 ^a^	±1.39	10.8 ^b^	±0.52
12:0, Lauric Acid	5.7	±1.33	5.2	±1.74	5.6	±0.66
14:0, Myristic Acid	11.7	±1.81	10.8	±1.85	11.8	±0.56
16:0, Palmitic Acid	26.3 ^b^	±2.05	23.2 ^a^	±1.97	26.9 ^b^	±1.07
18:0, Stearic Acid	8.5	±2.66	10.6	±3.42	8.3	±1.38
20:0, Eicosanoic Acid ^+^	0.82 ^a^	±0.14	0.98 ^b^	±0.23	0.86 ^ab^	±0.10
22:0, Docosanoic Acid	0.09 ^b^	±0.02	0.10 ^b^	±0.03	0.06 ^a^	±0.01
Σ Saturated Fatty Acids (SFA)	69.8 ^b^	±1.91	66.7 ^a^	±3.50	71.22 ^b^	±1.17
Odd and Branched-Chain Fatty Acids						
11:0, Undecanoic Acid	0.13	±0.04	0.10	±0.06	0.10	±0.02
13:0, Tridecanoic Acid	0.13 ^b^	±0.02	0.11 ^a^	±0.03	0.10 ^a^	±0.01
*iso* 14:0	0.16 ^c^	±0.02	0.13 ^b^	±0.03	0.10 ^a^	±0.01
*iso* 15	0.23	±0.02	0.24	±0.05	0.25	±0.03
15:0	1.14 ^c^	±0.09	1.07 ^b^	±0.08	0.91 ^a^	±0.05
*anteiso* 15:0	0.67 ^b^	±0.09	0.63 ^b^	±0.03	0.54 ^a^	±0.05
*iso* 16:0	0.34 ^c^	±0.05	0.29 ^b^	±0.08	0.25 ^a^	±0.02
*iso* 17:0	0.35	±0.03	0.37	±0.07	0.39	±0.03
17:0	0.70 ^b^	±0.11	0.75 ^b^	±0.18	0.57 ^a^	±0.06
*anteiso* 17:0	0.82	±0.21	0.76	±0.08	0.72	±0.08
17:1n-8	0.33 ^b^	±0.04	0.32 ^b^	±0.05	0.22 ^a^	±0.02
Σ Branched Chain Fatty Acids (BCFA)	2.6 ^b^	±0.23	2.4 ^b^	±0.18	2.2 ^a^	±0.13
Σ Odd Chain Fatty Acids (OCFA)	2.1 ^b^	±0.12	2.0 ^b^	±0.15	1.7 ^a^	±0.08
Σ Odd and Branched Chain Fatty Acids (OBCFA)	4.7 ^c^	±0.24	4.4 ^b^	±0.32	3.9 ^a^	±0.17
Monounsaturated Fatty Acids						
14:1, Miristoleic Acid	0.28	±0.21	0.22	±0.14	0.20	±0.07
16:1 c7+9, Palmitoleic Acid	0.79	±0.31	0.64	±0.19	0.61	±0.13
18:1 t9, Elaidic Acid (EA)	0.29 ^a^	±0.05	0.41 ^b^	±0.09	0.48^c^	±0.06
18:1 t11, *trans*-Vaccenic Acid	1.0	±0.38	1.4	±0.22	1.2	±0.41
18:1 c9, Oleic Acid (OA)	18.9 ^a^	±1.60	21.1 ^b^	±3.42	17.4 ^a^	±0.80
18:1 c11, cis-Vaccenic Acid	0.17 ^a^	±0.08	0.25 ^b^	±0.09	0.26 ^b^	±0.05
20:1n-9, Gondoic Acid	0.04 ^a^	±0.01	0.04 ^a^	±0.01	0.06 ^b^	±0.01
Σ Monounsaturated Fatty Acids (MUFA)	21.9 ^a^	±1.58	24.4 ^b^	±3.13	20.5 ^a^	±0.97
Polyunsaturated Fatty Acids						
18:2n-6 t9t12	0.20 ^a^	±0.06	0.26 ^b^	±0.04	0.24 ^ab^	±0.04
18:2n-6 c9t12	0.11 ^a^	±0.01	0.17 ^b^	±0.07	0.14 ^ab^	±0.04
18:2 c9c12, Linoleic Acid	2.0 ^a^	±0.27	2.6 ^b^	±0.29	3.1 ^c^	±0.22
18:3n-6, γ-Linolenic Acid (GLA)	0.03	±0.01	0.04	±0.02	0.04	±0.00
18:3n-3 c9c12c15, α-Linolenic Acid (ALA)	0.72 ^b^	±0.11	0.81 ^b^	±0.10	0.41 ^a^	±0.11
20:2n-6, Eicosadienoic Acid	0.07 ^b^	±0.01	0.07 ^b^	±0.02	0.06 ^a^	±0.01
20:3n-6, Dihomo-γ-Linolenic Acid (DGLA)	0.03 ^a^	±0.01	0.03 ^a^	±0.01	0.04 ^b^	±0.01
20:4n-6, Arachidonic Acid (AA)	0.15 ^a^	±0.01	0.17 ^b^	±0.02	0.18 ^b^	±0.02
20:5n-3, Eicosapentaenoic Acid (EPA)	0.10 ^b^	±0.03	0.10 ^b^	±0.02	0.06 ^a^	±0.03
22:5n-3, Docosapentaenoic Acid (DPA)	0.18 ^b^	±0.04	0.17 ^b^	±0.03	0.11 ^a^	±0.03
n3	1.0 ^a^	±0.14	1.1 ^a^	±0.12	0.6 ^b^	±0.15
n6	2.6 ^a^	±0.35	3.4 ^b^	±0.27	3.8 ^c^	±0.27
n6/n3	2.6 ^a^	±0.27	3.1 ^a^	±0.35	6.5 ^b^	±1.45
Σ Polyunsaturated Fatty Acids (PUFA)	3.6 ^a^	±0.46	4.4 ^b^	±0.31	4.4 ^b^	±0.31

^a, b, c^ = values in the same row that have a different superscript are significantly different at *p* ≤ 0.05, ANOVA and Student-Newman-Keuls post-hoc test. *LI-O and HI-C consisted in one sample in March, two samples per month from April to September and one sample in October (1 + 2 × 6 + 1), meanwhile LI-C consisted in two samples in April, one sample per month from May to September and two samples in October (2 + 1 × 5 + 2).+ This peak can include CLA c9t11, accounting for 0.6% of total FA in hay fed goat milk as reported by Bernard et al. [31].

**Table 3:** ANOVA of the Principal Components (PCs) eigenvalues. Data are reported as mean ± SEM.

Principal Component	Farm						
	Low Input-Organic		Low Input-Conventional		High Input-Conventional		
	Mean	SEM	Mean	SEM	Mean	SEM	P
**PC-1**	−0.47	1.05	2.04	1.31	−0.84	1.06	0.2123
**PC-2**	3.57 ^a^	0.40	1.23 ^b^	0.50	−4.36 ^c^	0.40	<0.0001

^a, b, c^ = values in the same row that have different superscript are significantly different at *p* ≤ 0.05, ANOVA and Student-Newman-Keuls post-hoc test.
